# Anatomical Connectivity Influences both Intra- and Inter-Brain Synchronizations

**DOI:** 10.1371/journal.pone.0036414

**Published:** 2012-05-10

**Authors:** Guillaume Dumas, Mario Chavez, Jacqueline Nadel, Jacques Martinerie

**Affiliations:** 1 Université Pierre et Marie Curie-Paris 6, Centre de Recherche de l'Institut du Cerveau et de la Moelle épinière, UMR-S975, Paris, France; 2 Inserm, U975, Paris, France; 3 CNRS, UMR 7225, Paris, France; 4 ICM – Institut du Cerveau et de la Moëlle épinière, Paris, France; 5 Centre émotion, CNRS, USR 3246, Paris, France; Technical University of Madrid, Italy

## Abstract

Recent development in diffusion spectrum brain imaging combined to functional simulation has the potential to further our understanding of how structure and dynamics are intertwined in the human brain. At the intra-individual scale, neurocomputational models have already started to uncover how the human connectome constrains the coordination of brain activity across distributed brain regions. In parallel, at the inter-individual scale, nascent social neuroscience provides a new dynamical vista of the coupling between two embodied cognitive agents. Using EEG hyperscanning to record simultaneously the brain activities of subjects during their ongoing interaction, we have previously demonstrated that behavioral synchrony correlates with the emergence of inter-brain synchronization. However, the functional meaning of such synchronization remains to be specified. Here, we use a biophysical model to quantify to what extent inter-brain synchronizations are related to the anatomical and functional similarity of the two brains in interaction. Pairs of interacting brains were numerically simulated and compared to real data. Results show a potential dynamical property of the human connectome to facilitate inter-individual synchronizations and thus may partly account for our propensity to generate dynamical couplings with others.

## Introduction

What causes the propensity of human brains to generate dynamical couplings is still currently an intriguing question. One candidate explanation could be related to connectivity properties of the brain. In an attempt to explore this hypothesis, the aim of the present study was to compare simulated interacting brains to real brains of interacting partners. The modern scientific panoply gathering together connectomics with hyperscanning techniques makes now such an attempt available. The question of an intrinsic relationship between structure and dynamics however is not new. In complex systems research, the complementariness between structure and dynamics has been a long lasting topic. Indeed, while structure shapes the dynamics by providing constraints, dynamics modifies the structure itself by adding plasticity. In cognitive sciences, the coordination dynamics of brain and behavior has been an early feature of experimental and theoretical work [1], [2]. Recently, the structure∼function coupling has attracted the attention of neuroscientists, insofar as both structure and dynamics contribute to the evolution of the nervous system via their mutual coordination [3], [4]. Complex network theory [5], [6] combined with the increasing amount of data gathered by structural and functional neuroimaging techniques [7], [8] is well represented by the field of connectomics [9], [10] which has been devoted to reconstruct the whole nervous system network with histological and more recently neuroimaging techniques such as Diffusion Tensor Imaging (DTI). Various studies in this nascent domain have revealed complex network topology in the physical scaffolding of the brain [11], [12].

Thus on one hand, neuroimaging techniques can give access to brain networks as well as to brain dynamics, and on another hand, neurocomputational approaches provide tools that allow combining the two data sources and investigating their complementariness. As an example, the “Virtual Brain” approach [13], [14], offers a test bed for theoretical models inherited from experimental observation. Recent works have illustrated the predictive power of such approach by simulating the anti-correlated BOLD functional network [15] and the multistable attractor landscape [16] observed during resting state.

Despite the growing interest for whole-brain simulation, these techniques have only been applied so far to the comparison with isolated brains. To-date however, comparisons can be lead between simulated interacting brains and real brains recorded during an ongoing interaction. Hyperscanning techniques allow such dual recordings [17], [18]. Pioneer works in fMRI have demonstrated that inter-brain relationships appear between brain activity of subjects immersed in the same perceptual context [19] or engaged in an economical game [20]. Later explorations have extended these observations to social communication [21], [22].

Hyperscanning-EEG have opened the explorations to the millisecond time-scale [23], [24]. This is particularly worthy since neural synchronizations have been proposed as a plausible mechanism for a large-scale integration of cognitive information in the brain [25], [26]. Recently, our team has demonstrated with EEG-hyperscanning that neural synchronizations can also be observed between the brains of two persons engaged in a reciprocal social interaction [27]. Our objective in the present paper was to investigate the effect of the individual anatomical connectivity on the inter-individual functional connectivity. The main issue was to quantify to what extent inter-individual synchronizations are related to the anatomical and functional similarity of the two brains in interaction. Within this framework, we designed whole brain numerical simulations combining a connectome dataset [28], [29] with a revisited version of the Kuramoto model of weakly coupled oscillators. The model was first validated at the intra-individual level via a forward modeling and a statistical comparison with real resting state data. Then, we created simulated inter-individual interactions between pairs of virtual brains. The simulation used an artificial sensorimotor coupling by linking the motor regions of each brain with the visual regions of the other [30]. This simulation allows quantify the influence of brain anatomy on the dynamical similarity of the two brains and evaluate its potential role in the emergence of inter-subject sensorimotor couplings.

## Materials and Methods

### Experimental Data

The experimental data used in this paper are taken from our former study about inter-brain synchronizations [27] where 18 participants paired as 9 dyads were recorded simultaneously with dual-video and dual-EEG setups while they were engaged in spontaneous imitation of hand movements.

#### Apparatus and setting

**Figure 1:**
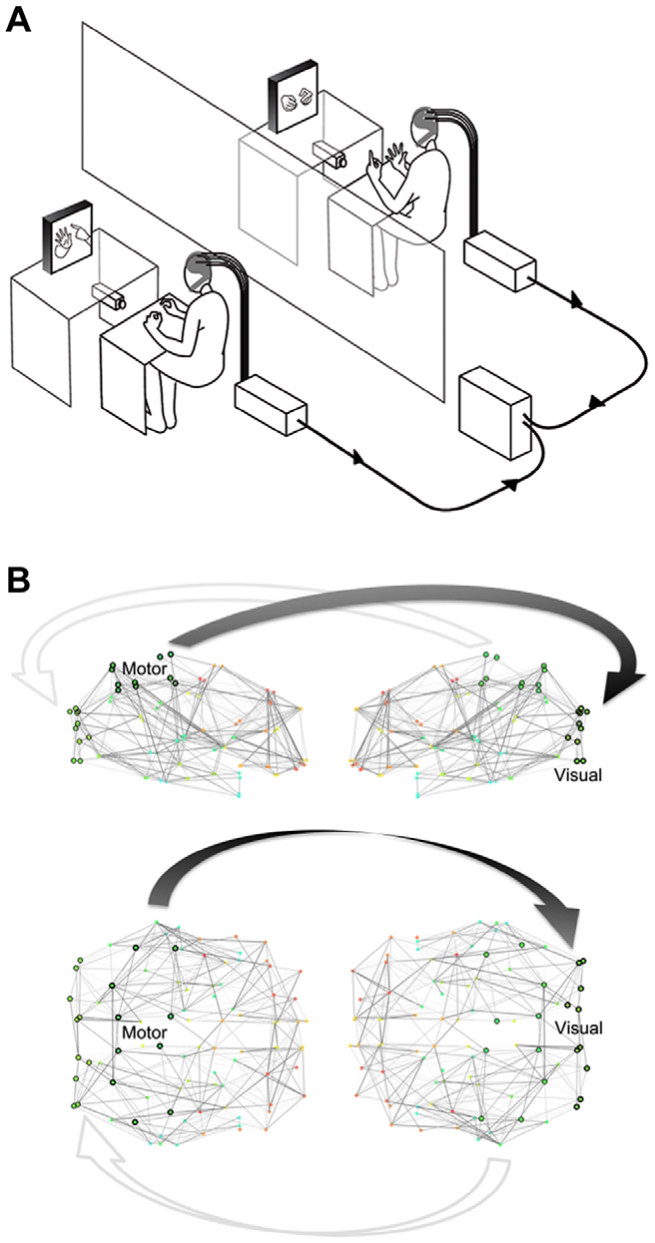
Experimental and simulation setups. (A) Apparatus and experimental setting of the double video system and dual-EEG recording [27]. (B) Right and Top views of the pair of virtual brains. Each weighted network represents the 90 brain regions and their average anatomical connectivity. Arrows indicate the directed coupling from the motor to visual.

The experiment was conducted in two separate laboratory rooms. The design and equipment were similar to the double-video system designed by Nadel and colleagues for their developmental studies of sensitivity to social contingency in infants [31], except that a dual EEG recording system was added to the setup (Fig. 1A).

#### Protocol

The protocol was composed of different conditions. Here, we used the control condition where subjects were asked to rest without seeing each other, and the spontaneous imitation condition where subjects were able to see each-other’s hands and moved their hands freely. In this second condition, the instruction was to continuously move the hands and imitate the other at will. All the movements were bi-manual and intransitive (meaningless gestures). Each session would begin with a 15 seconds long resting state recording followed by 15 seconds where subjects started to move their hands without seeing each-others, and then a spontaneous imitative interaction of 90 seconds. Behavior during spontaneous imitation was controlled by a frame-by-frame video analysis. The subjects had to move continuously their hands and adopted a balanced repartition of the roles (model or imitator) during their interaction. It assessed that subjects moved continuously their hands and adopt a balanced repartition of the roles (model or imitator) during their interaction (See [27] for details).

#### Recordings

The simultaneous neural activities of the two subjects were recorded with a dual-EEG recording system. This system was composed of two Acticap helmets with 32 active electrodes each. The ground electrode was placed on the right shoulder of the subjects and the reference was fixed on the nasion. The impedances were maintained below 10 kΩ. Data acquisition was performed using a 64-channels Brainamp MR amplifier from the Brain Products Company (Germany). Signals were analog filtered between 0.16 Hz and 250 Hz, amplified and digitalized at 500 Hz with a 16-bit vertical resolution in the range of +/−3.2 mV. Spatial positions of the electrodes were recorded with a Polhemus system for all subjects.

#### Pre-processing

Four electrodes were excluded from the analysis because of too low signal to noise ratio. The correction of eye blink artifacts in the remaining EEG data was performed using a classical Principal Component Analysis (PCA) filtering algorithm [32]. We used 800 ms windows with 400 ms of overlap. EEG signals were then controlled visually in order to discard periods with remaining artifacts. These were excluded from the analysis and, in order to avoid border artifacts induced by their suppression, we smoothed the joints by a convolution with a half-Hanning window. This operation may impact low-frequency part of the signal but the spectral characteristics of the contamination have no overlap with the frequency bands investigated here.

### Computational Model

#### Structural connectivity

In order to represent the two brains of the virtual partners we used two connectomes (Fig. 1B). We took the connectivity matrix obtained as described in [28], [29]. The elements of this matrix describe the probabilities of connection between the 90 regions of the Tzourio-Mazoyer (TZ) brain atlas [33]. This matrix of anatomical connectivity (Fig. S1D) was generated by averaging Diffusion Magnetic Resonance Imaging (DW-MRI) data over 20 participants. As these probabilities are related to the density of fibers, they represent an approximation of the connection strength between each pair of brain regions.

Each matrix (See Fig. S1D) was embedded as a spatial weighted and non-oriented graph were the positions in the Montreal Neurological Institute (MNI) coordinates of each region were taken as the barycentre of all the voxels of the region in the Automated Anatomical Labeling (AAL) atlas [33] (See Fig. S1 A, B & C).

Shuffled intra-individual versions of the connectome were created by permuting the connectivity matrix while keeping it symmetric and with zeros over the diagonal.

#### Dynamical modeling

The dynamical model is adapted from the multiple oscillators model of Kuramoto [34] which is the most common model in the study of synchronization phenomenon in physics [35]. Furthermore, this model is thought to be a plausible approximation of neurobiological oscillating processes [36]–[38], specifically when the couplings between oscillators are extracted from real anatomical connectivity data [15], [39]. Here each brain region was represented as an oscillator in the gamma frequency band since this rhythm has been associated with the local processing of information at small scale [40], [41] and with the integration of neural information at large scale [25], [42].

The model equations read as:
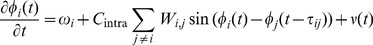
(1)where 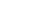 stands for the phase of the i^th^ oscillator at the time t, 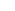 is the natural frequency of the i^th^ oscillator taken randomly from a normal distribution centered on 40 Hz and with a standard deviation of 8 Hz; C_intra_ is a control parameter related to the scaling of the global anatomical connectivity (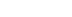); 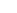 is the propagation delay between the i^th^ region and the j^th^ region based on Euclidean distance between the two nodes multiplied by a standard axonal velocity of 1.65 m.s^−1^ [43]; 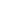 is a dynamical random perturbation such that 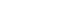 and 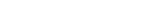 with 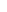 the Dirac function. In the present work *D* is considered equal to 0.1; and W_i,j_ is the coupling parameter between the i^th^ and j^th^ regions based on the connectivity dataset. The simulations were run over 5000 samples using the Euler technique at a sampling frequency of 500 Hz, i.e. dt = 0.02. They were initialized with random phases. The 1000 first transitory samples were discarded from the analysis.

#### Modeling sensorimotor coupling

In order to model the two virtual partners, we created a 180-squared matrix W with two blocks of 90 Regions Of Interest (ROI) for each virtual brain (See Fig. S1D). Thus, region 91 corresponds to region 1 of brain 2. W also integrates inter-individual coupling elements between the motor regions of each partner and the visual regions of the other, and vice-versa, thus simulating the sensorimotor coupling at play during a behavioral interaction [30]. The selected motor regions were left and right paracentral lobules (TZ n°: 69, 70), left and right post-central areas (TZ n°: 57, 58), left and right parietal areas (TZ n°: 59, 60, 61, 62) and left and right precuneus (TZ n°: 67, 68). The selected visual regions were left and right calcarine areas (TZ n°: 43, 44), left and right cuneus (TZ n°: 45, 46) and left and right occipital areas (TZ n°: 49, 50, 51, 52, 53, 54).

The W matrix was modified as follows:
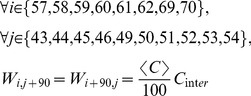
(2)where 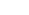 is equal to the average coupling parameter of the connectivity dataset.

C_inter_ is thus similar to the previous mentioned scaling parameter C_intra_ but is related to the intensity of informational influence existing in reality between the agent’s action and the partner’s action perception (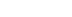). Here we assumed that the main part of the sensorimotor stream of information is conveyed through the agent motor areas to the observer’s visual areas. The delay for this inter-individual coupling was taken as null since the causal influence of the behavioral interaction is mediated by photons through the dual-video system. Brain-muscles and retina-brain delays were not taken into account in the present study. Additionally, we did not simulate other mechanisms potentially at play in the sensorimotor coupling such as those linked to the proprioceptive representation of own movement by each partner.

To assess the role of anatomical individual connectivity on inter-subject interactions, we added two types of simulations using shuffled versions of the connectome: one where paired brains shared the same shuffled connectome, and the other where each brain had a different shuffled version. This aimed at quantifying the effect of the anatomical structure at both intra- and inter-individual levels.

#### Numerical simulations

The different simulations were generated for 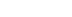 (step = 0.01) and 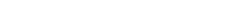. All simulations used different sets of pulsations for the two virtual brains (i.e. the 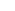 in Equ. 1) and the initial state of phases was taken from a uniform random distribution between −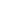 and +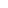.

Programming was done with Matlab (RC2009b, The MathWorks). The Graphical Processing Unit (GPU) implementation used the GPUmat toolbox (http://gp-you.org/) for the implementation on GPU.

### Forward Model

In order to compare real and simulated EEG data, we computed with the Brainstorm Matlab toolbox [44] a forward model with the overlapping sphere technique. The model was done on the anatomical MNI template Colin27 [45] after repositioning the electrodes according to the average spatial positions across real subjects recorded with a Polhemus system. It gave us a gain matrix G referring to the virtual EEG signal that could be observed at the scalp level in function of the activity of the cortical sources.

Thus we obtained EEG = G*S, where G stands for the gain matrix of the forward model and S for cortical source signals. Cortical sources are modeled as 10000 elementary dipoles located at the vertices of the cortical mesh surface, pointing outward the surface (i.e. 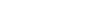 and 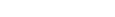). The signal for each source *k* is obtained by applying a cosinus function to the phases of the nearest region *i* of the TZ atlas localized in the MNI space: 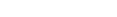.

### Dynamical Measurements

Synchronization is usually measured in Kuramoto systems with the order parameter. Its value quantifies the phase coherence across oscillators at a given point in time. It vanishes when their phases are uniformly distributed and converges to 1 when they become aligned. Here we computed the time-averaged order parameter of each virtual brain following the formula:
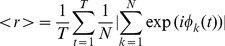
(3)where k is the region of the considered virtual brain, N = 90 regions and T = 4000 samples = 8s.

The EEG dynamics was then quantified with the Phase Locking Value (PLV) which provides a frequency-specific synchronization measure between two signals across time [46] and is commonly used in EEG/MEG studies [25]. Similarly to the order parameter, PLV is null when phases’ differences between two signals are uniformly distributed over time and approaches 1 when they become constant. After a band pass filtering of the scalp signal in the gamma range between 32 and 48 Hz, we applied a Hilbert transform to extract the instantaneous phase 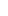 of each signal. The PLV formula for two channel p and q is given by:
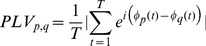
(4)where T is the number of samples considered in each time window and | | the absolute value. PLV matrices were generated by averaging 10 non-overlapping 800 ms windows.

In the following, we will use PLV when the two electrodes are taken from the same subject and h-PLV (or hyper-PLV) when the two electrodes belong each to a different subject.

### Similarity with Real Data

**Figure 2:**
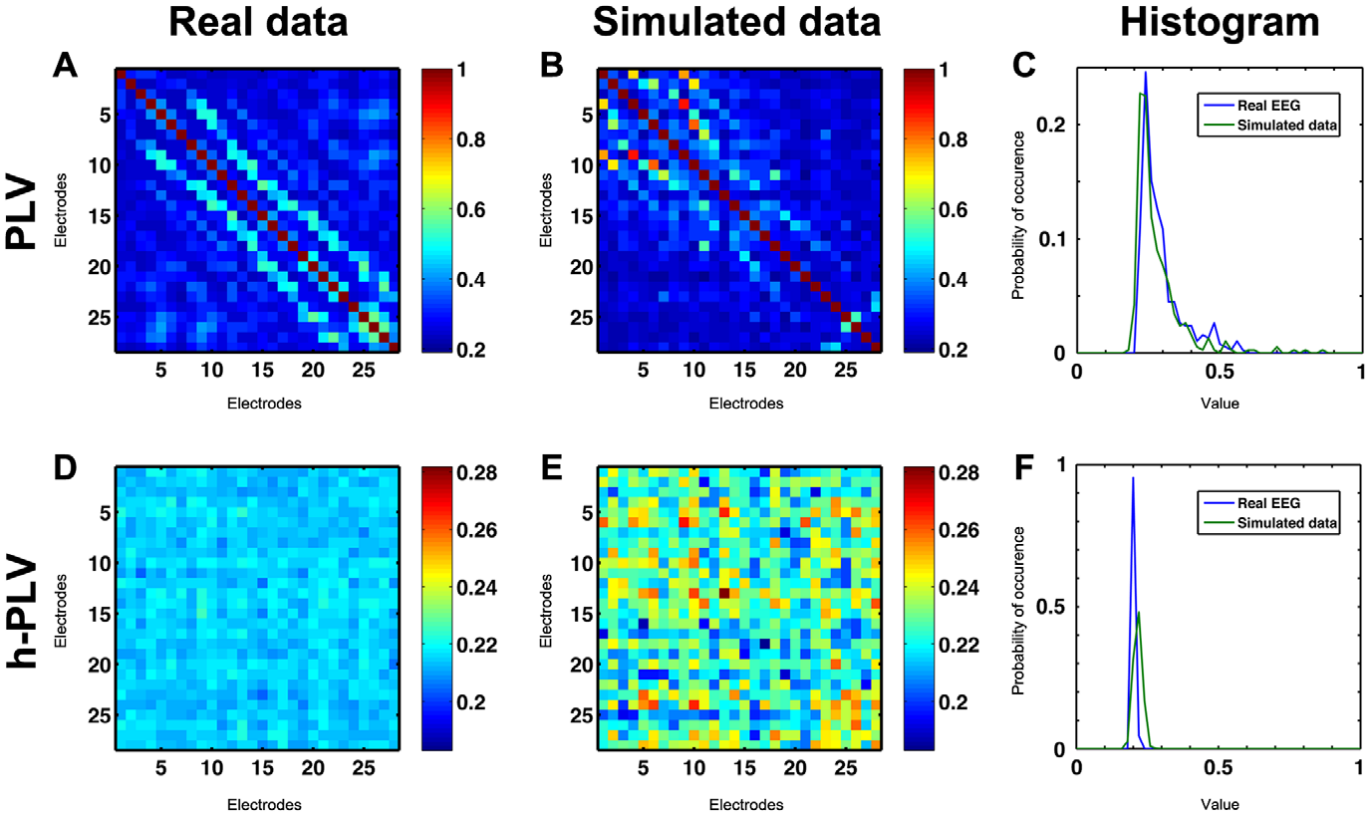
Real and simulated functional data. PLV matrices for real (A) and simulated (B) data and related histogram (C). h-PLV matrix for real (D) and simulated (E) data and related histogram (F). PLV and h-PLV are computed for the gamma band and averaged across either the 9 pairs of real subjects during resting state condition or 9 pairs of simulated subjects with C_intra_ = 0.49 and C_inter_ = 0. It can be seen from this example that PLV and h-PLV exhibit different distributions. Notice that the difference of the dynamics between the partners gives an asymmetry in the h-PLV matrix.

The dissimilarity distances between simulated and real data were calculated with the Mahalanobis distance [47] for PLV and h-PLV matrices respectively (See Fig. 2 for average matrices and distributions).

This distance is defined with the formula:
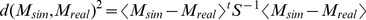
(5)where the 18 rows of M_sim_ and M_real_ matrices represents all simulated/real subjects and the 784 columns are the PLV/h-PLV values for all pairs of electrodes; 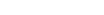 is the average difference between the two populations and S represents their pooled covariance matrix. This distance thus takes into account first and second order statistical parameters of the data. Via the comparison between the simulations with real anatomy and their shuffled versions, we aimed at emphasizing the area of the parameter space where the model expressed the most realistic dynamics.

We first sought the best fitting area of our model at the intra-individual level by setting C_inter_ = 0 and compared PLV matrices with those of the real resting state condition. We also found the best fitting region at the inter-individual level by comparing both PLV and h-PLV matrices with those of the behavioral interaction condition.

**Figure 3:**
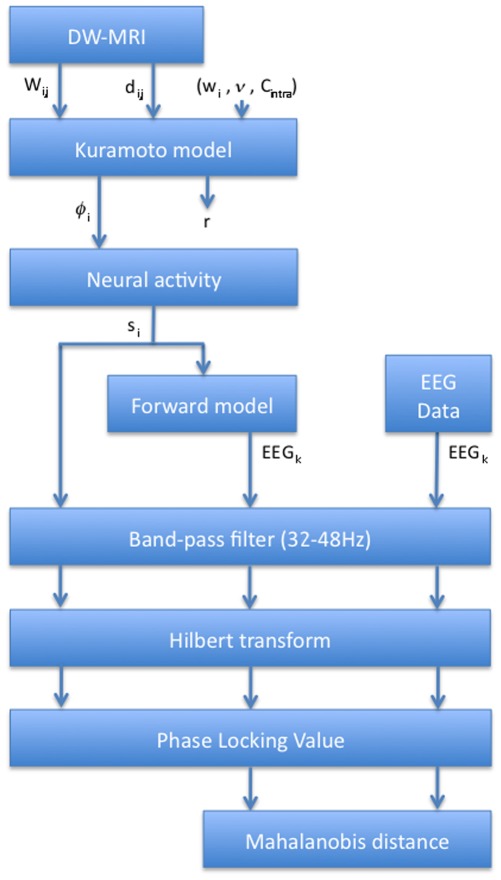
Procedure Flowchart illustrating the different steps of the simulations and their comparisons with the real EEG data.

For clarity, all the different steps of the above procedure are represented in the flowchart shown in Fig. 3.

## Results

The results section is composed of two main parts. The intra-brain part tests the effect of the strength of the anatomical coupling on the oscillatory activity within each of the two separated virtual brains. This intra-brain analysis aims at finding the more realistic interval of the modeled parameter space before moving at the inter-brain level. The inter-brain part tests the effect of the real anatomical structure on the sensorimotor coupling between two virtual interacting partners.

### 1. Anatomical Influence on the Intra-individual Functional Connectivity

For no anatomical coupling (C_intra_ = 0), all oscillators were independent and the same distribution of frequency was observed at both ROIs and scalp levels. Then, while the strength of the anatomical connectivity increased, clustering was observed between ROIs.

**Figure 4:**
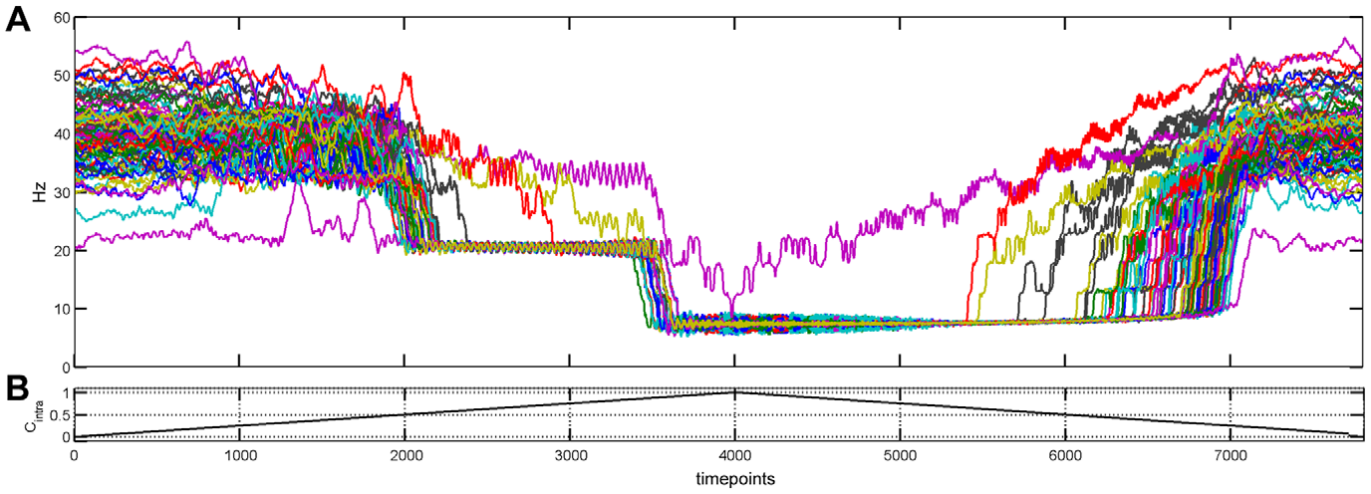
Example of simulation with variation of the C_intra_ control parameter over time. (A) Timecourses of all ROIs instantaneous frequency. (B) Related timecourse of the C_intra_ parameter.

**Figure 5:**
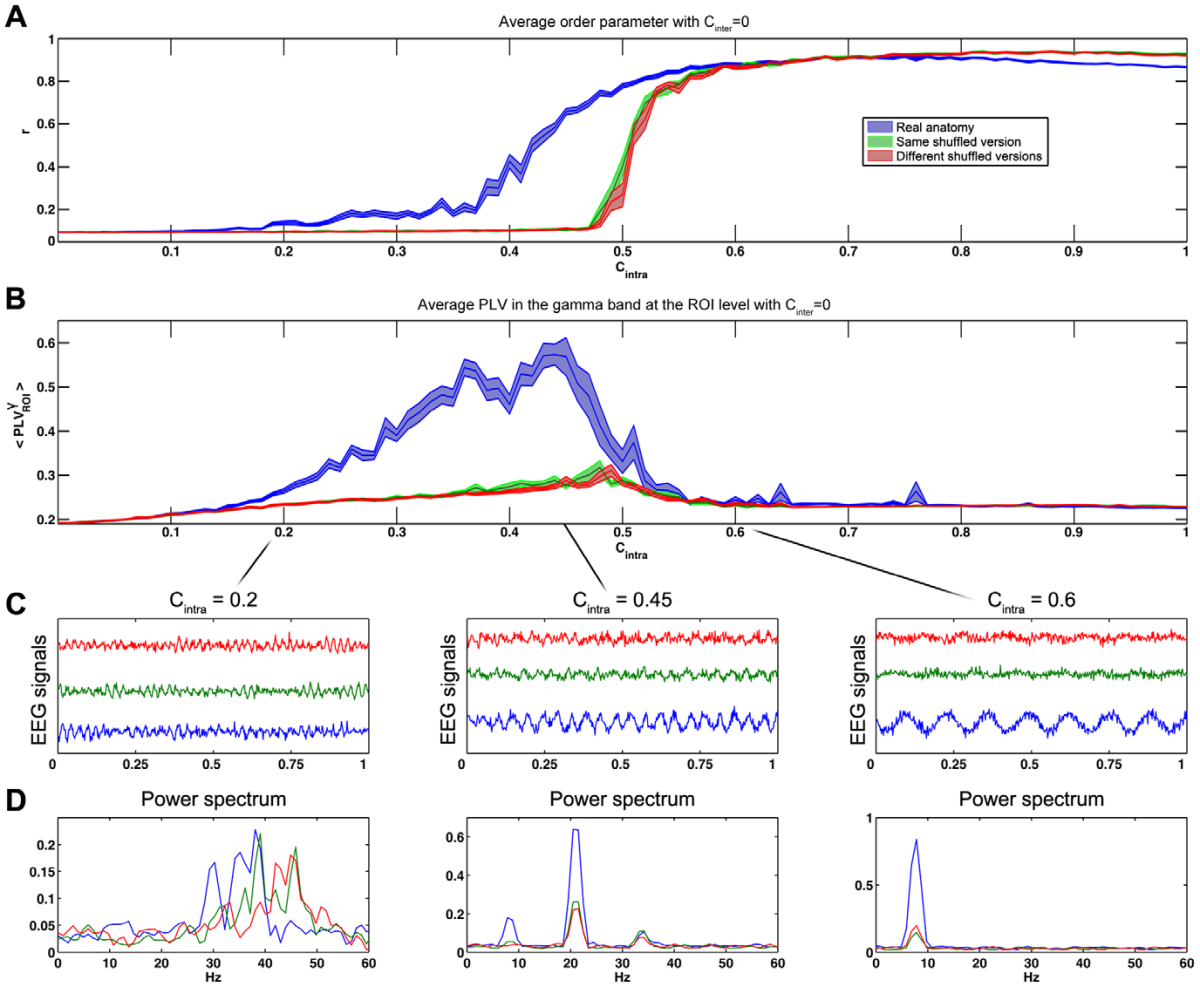
Influence of the global anatomical connectivity strength on the model’s dynamics. (A) Average order parameter across the 90 ROIs. Influence of the global anatomical connectivity strength at the ROIs level. Results are averaged across 18 simulations with C_inter_ = 0. Areas stand for the standard error. Blue: real connectivity. Green: identic shuffled connectivity for the two virtual brains in same dyads. Red: different shuffled connectivity for all virtual brains. (B) Average PLV in the gamma band between all the ROIs inside each virtual brain. (C) Example of simulated EEG signals. (D) Power spectrum for each EEG signals of C.

As expected, a phase transition was then assessed at the ROIs level by a change on the order parameter while the anatomical strength increased (Fig. 4A). This transition occurred lately and sharply for the shuffled versions of the connectome (Fig. 5A). In all cases, the transition was characterized by an increase of the average PLV values in the gamma band (Fig. 5B). The two shuffling strategies did not make any differences at the intra-individual level. They both showed a weaker increase in the average PLV values than the real connectome.

During this transition, intermediate beta rhythms peaks (between 21 Hz and 34 Hz) first appeared transiently around C_intra_ = 0.45 (Fig. 5D). Then, an alpha-like low frequency rhythm appeared and shrank the gamma rhythms (Fig. 4 and Video S1). The period of this emergent rhythm was linked to the axonal velocity and to the size of the connectome (Fig. S2). The observed proportionality suggests that the period of this low-frequency rhythm corresponds to the average back and forth propagation time across the connectome.

**Figure 6:**
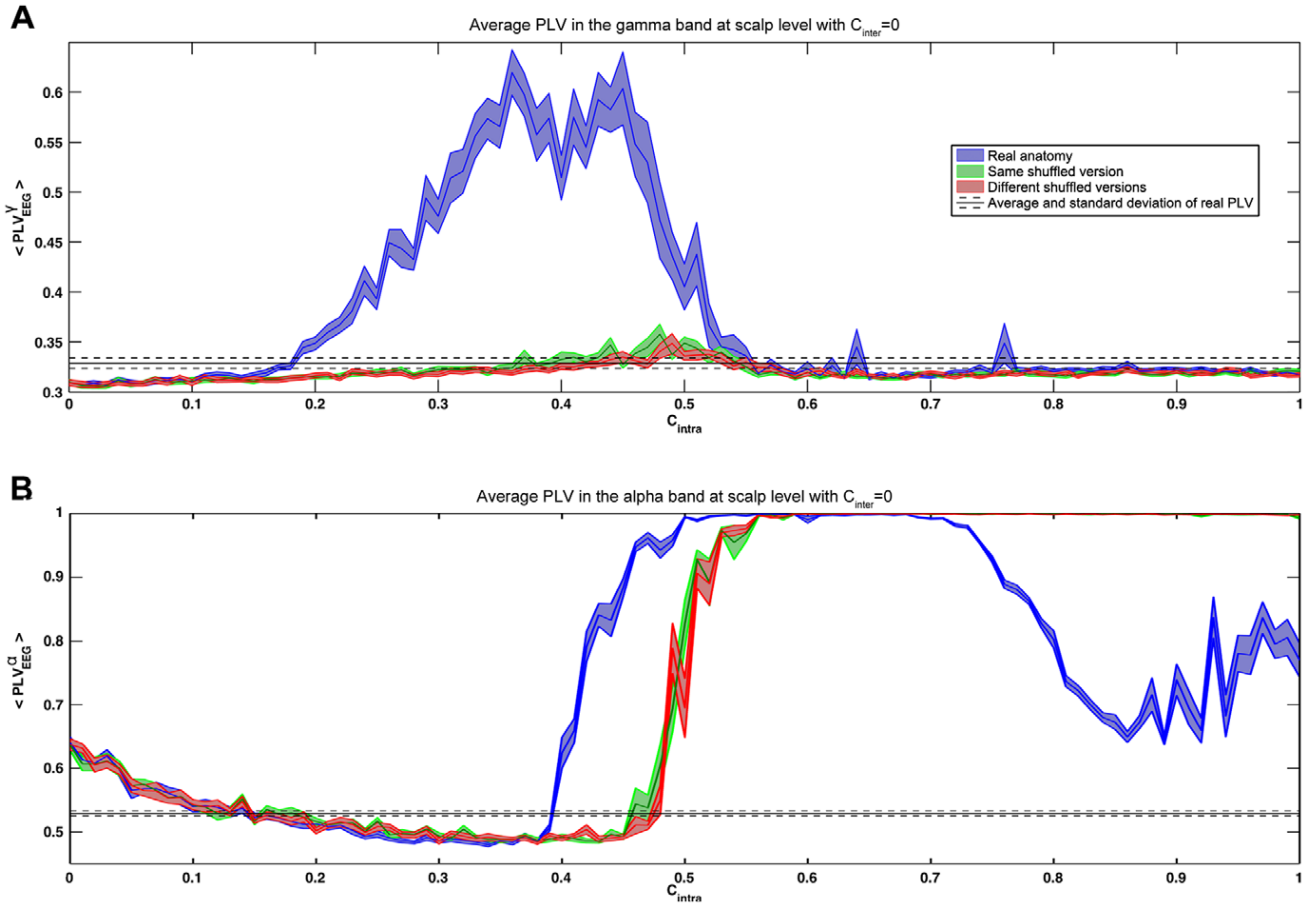
Influence of the global anatomical connectivity strength on intra-brain synchronization. Average PLV across all pairs of electrodes inside each simulated subject helmet for the gamma (A) and alpha (B) frequency bands. The decrease of PLV after C_intra_ = 0.7 for the alpha band seems caused by fluctuations of the mean low-frequency rhythm peak at strong anatomical coupling.

A similar transition phenomenon was observed at the scalp level (Fig. 6A): PLV collapsed after the transition in the gamma frequency band and increased in the alpha frequency band (Fig. 6B).

**Figure 7:**
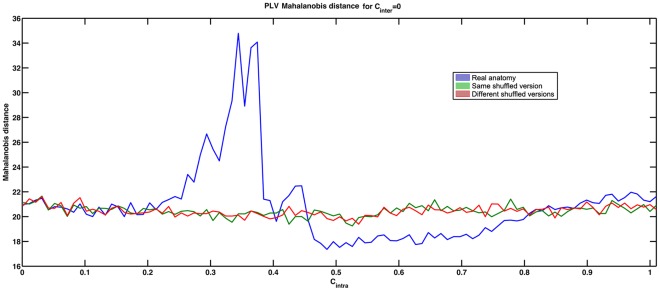
Mahalanobis distances between simulated and real resting state data based on PLV matrices in the gamma band.

We compared the simulated EEG signals with those of real resting state data (C_inter_ = 0). The dissimilarity distance between simulated and real resting data was reached in an interval of the later part of this phase transition. In this interval (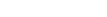), the distances obtained for the simulations with the real connectome were smaller than those obtained with the shuffled versions of the connectome (Fig. 7).

### 2. Anatomical Influence on the Inter-individual Functional Connectivity

**Figure 8:**
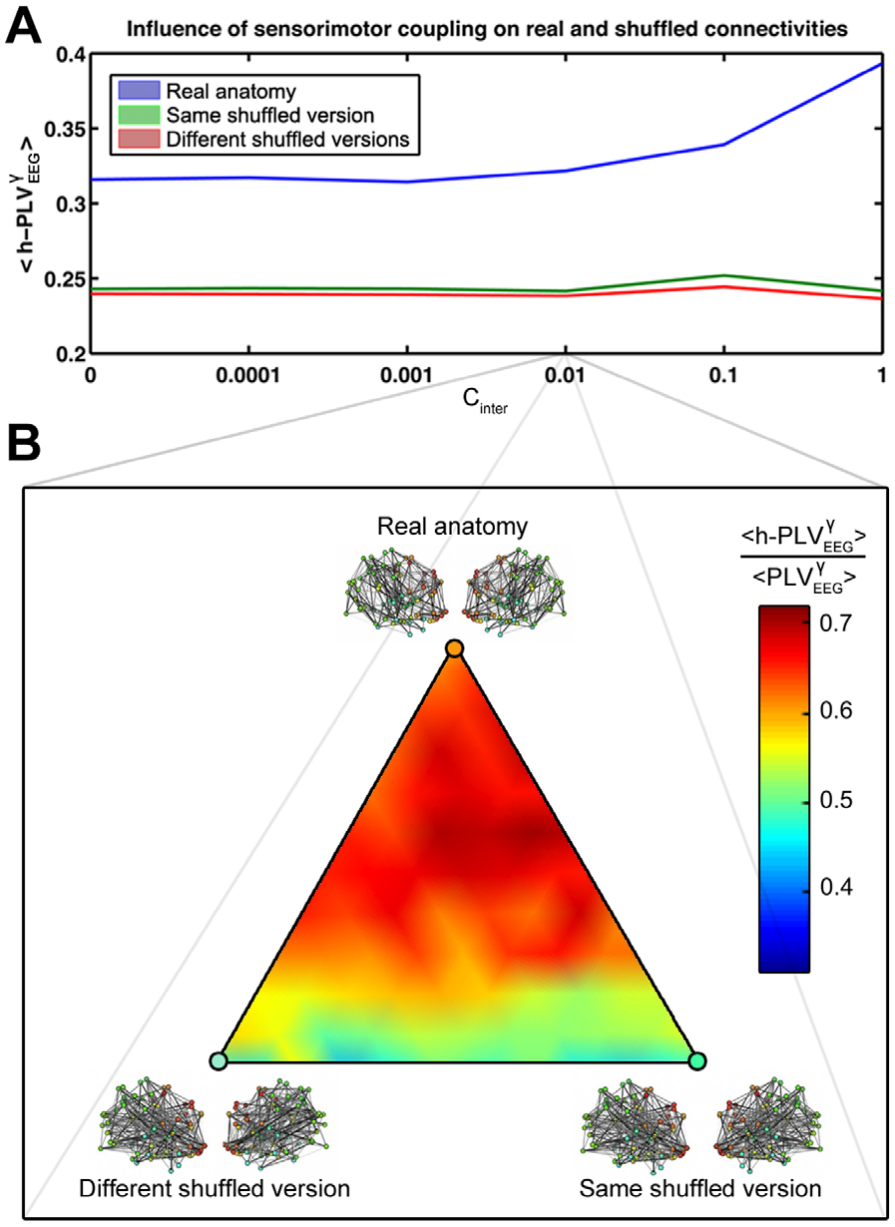
Influence of the anatomical connectivity on inter-brain synchronization. (A) Average response of the artificial sensorimotor coupling strength on h-PLV across the best fitting area (C_intra_ between 0.5 and 0.6). (B) Effect of the anatomical topology and similarity on the normalized h-PLV for C_inter_ = 0.01. Each point is computed for a normalized linear combination of the three cases: same real anatomy, same shuffled version and different shuffled versions.

In the simulated resting state (C_inter_ = 0), the h-PLV between the two virtual brains were not null and these “residual synchronizations” increased as a function of the strength of the anatomical connectivity before the phase transition and then collapsed for C_intra_ = 0.45 as for the PLV (Fig. S3). After the phase transition - i.e. the area matching at best the real data (Fig. 7) - the average h-PLV increased as the strength of the artificial sensorimotor coupling C_inter_ was incremented between the two virtual partners (Fig. 8A). It is worth noticing that simulations with different shuffled versions of the connectome did not show this effect. Nevertheless, the average h-PLV was slightly higher for virtual brains sharing the same shuffled version of the connectome than for those with different shuffled versions. For a given C_inter_ value, the anatomical connectivity (i.e. real or shuffled anatomy) appeared to have a stronger effect on h-PLV than the similarity between the connectivity of the two virtual brains (Fig. 8B).

## Discussion

Research has already demonstrated that the biological structure of the human brain offers a rich panel of dynamical states with global efficiency [48] and information processing enhancement [49]. Here, we focused on how the anatomical structure of the brain facilitates the internal processing of cognitive information and the ability to generate inter-individual coupling via perception and action [50]–[52]. More precisely, we aimed at investigating the influence of the anatomical connectivity on neural synchronizations at both intra- and inter-individual levels. We created a biophysical model integrating a real anatomical connectivity dataset based on the 90 ROIs atlas of Tzourio-Mazoyer [33]. These ROIs represent the structural level of the dynamical modeling based on the Kuramoto’s weakly coupled oscillators. The current study integrates realistic couplings based on the anatomical connectivity and delay proportional to the average spatial distances between ROIs and axonal velocity. The frequencies of the oscillators were fixed in the gamma band (between 32 and 48 Hz) as this brain rhythm is related to a neural processing at the local level [53], [54] and is correlated with the hemodynamic signal [55]. A recent model suggests that the spontaneous local gamma oscillations could also enhance a neural network selectivity and responsiveness to external inputs [56]. Furthermore, long range gamma phase synchronization has been proposed as a plausible mechanism for large-scale integration of neural information at the individual level [42] and were also observed in our former hyperscanning study between two brains [27]. As these recordings were at the scalp level, we applied a forward model to our brain simulation to create virtual EEG recordings compared to real EEG resting state data.

In order to quantify the inter-brain effect of the real anatomical structure, we used two different shuffling strategies on the connectome. The first strategy was to generate for each simulation a new shuffled version and use it on each pair of virtual partners. This allowed quantify the impact of simultaneously sharing anatomical structure and internal dynamics. The second strategy was to take a different shuffling for the two-paired virtual brains and thus measure the residual synchronization due to the sole similarity of internal dynamics.

### 1. Intra-individual Dynamics

The first part of our analysis focused on the individual brain where we tuned the gain of the anatomical connectivity (C_intra_) in order to fit the model with the real resting state data. At the level of the sources (ROIs), the order parameter gives access to a coarse synthesis of the dynamics by quantifying the spatial coherence of all oscillators. Two main states were observed by varying the anatomical coupling strength: firstly, the oscillators kept their high frequency and tended progressively to form clusters as the anatomical connectivity increased, secondly, a global coherent oscillatory state appeared in the low frequency band.

Interestingly, the transition between these two states was different in the real and in the shuffled cases (See Fig. 5): with the same structural connectivity strength, the real connectome was more efficient to synchronize the oscillators than the shuffled versions. This is consistent with past results pointing out the structural efficiency of small-world network to synchronize large number of oscillators [57]–[60]. However, the transition between the two dynamical states (desynchronized and synchronized) was also larger in the real anatomical case (see related C_intra_ intervals in Fig. 5A). The shape of the curve not only confirms that the structure of the brain allows a faster transition to coherent state, probably thanks to its “small-worldness” [4], [61], but it also suggests that the dynamics is relatively robust to structural fluctuations.

Computational models show a similar effect of the delay in enhancing both synchronization [62] and stability of the dynamics despite the existence of perturbations created by the environment [63]. A recent study by Perez and collaborators [64] has shown that the combination of a real network topology and of delays of axonal conduction creates a coherent spiking dynamics. Here, we observed this phenomenon after the phase transition interval where the dynamics converged to a very stable 8 Hz limit cycle similar to the alpha rhythm typically observed in electrophysiological studies. Consistent with previous findings about time delay in the Kuramoto model [65], this low-frequency rhythm corresponds to the average back and forth propagation across the whole connectome (Fig. S2). Such result suggests an effect of the global anatomical structure (connectivity) of the brain on the generation of neural rhythms.

In order to calibrate our model, we then used the Mahalanobis distance to quantify the dissimilarity between the PLV observed in real EEG data during resting state, and those reconstructed after forward modeling during a virtual resting state (C_inter_ = 0). A striking result was that the best fitting point (where the Mahalanobis distance reaches its minimum) occurred only for the simulations with the real connectome and was at the transition discussed above. In this interval (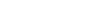), the real anatomical connectivity of the human brain enhances synchronization in high frequency band and makes emerge intrinsic rhythms in the low frequency band. The emergence of alpha-like oscillations disrupts the synchronized patterns in the gamma band. This state makes possible a mechanism of active desynchronization observed more than ten years ago by Rodriguez and colleagues [66] and interpreted as an “active uncoupling of the neural assemblies, necessary to proceed from one cognitive state to another”. Using a similar neurocomputational approach, a recent study has shown that the cortical activity at rest exhibits multistability. It has also explicitly demonstrated that the related attractor landscape is encoded in the neuroanatomical connectivity [16]. Interestingly, the authors emphasized that such phenomenon has also been reported with a biophysical corticothalamic model of alpha rhythm during rest [67]. Our results converge with these findings and points out a direct link between alpha rhythm frequency and the spatial embeddedness of the connectomes.

### 2. Inter-individual Dynamics

In the second part of the study we focused on the inter-individual synchronizations. We aimed at quantifying the synchronization that could be observed in the absence of any inter-individual interaction. These inter-brain “residual synchronizations” are the consequences of the similarities between the two individual brains - either real or simulated - at both structural and dynamical levels. When we compared the simulations using the real connectome to the simulations using the two shuffled strategies, we observed the facilitating role of the real anatomical connectivity in these “residual synchronizations”. Indeed, while the strength of the connectivity (C_intra_) increased, real versions of the connectomes tended to synchronize more than the shuffled versions. This suggests a potential dynamical property of thetopological brain structure to facilitate inter-individual residual synchronizations and thus may partly account for our propensity to generate dynamical couplings with others">This suggests a potential dynamical property of thetopological brain structure to facilitate inter-individual residual synchronizations and thus may partly account for our propensity to generate dynamical couplings with others. Interestingly, if the connectomes were shuffled, the fact that the two networks share or not the same structure had no apparent influence. There was nevertheless in both cases weak residual synchronizations caused by the dynamical similarity of all the oscillators (See Fig. 8).

Finally, we looked at the effect of sensorimotor coupling on the inter-brain synchronization. We focused on the best fitting region of the model and increased progressively the coupling between visual regions of each virtual brain with motor regions of the other. The effect of this coupling on the inter-brain synchronization was maximal for the real connectivity compared to shuffled versions (Fig. 8A). However, it appeared that this effect was absent outside the best fitting interval (Fig. S3A). Before the transition, the internal coupling is probably not sufficient to spread the information from the visual area to the remaining part of the virtual brain. After the transition, the h-PLV in the gamma band vanished despite a strong inter-individual coupling. Simultaneously, we observed the increase of h-PLV in the alpha rhythm (Fig. S3). Interestingly, the intra-individual synchronies within each virtual brain remain insensitive to the sensorimotor coupling between them (Fig. S3).

As already proposed, the anatomical functional similarity across humans could explain a tendency to enter in synchronization while immersed in the same perceptual context [68] or while doing the same perceptual-motor task [23]. Our results suggest that the similarity of endogenous dynamics (here the distribution of the frequency of the oscillators) altogether with the similarity of anatomical structure support this effect. They also suggest that the anatomical connectivity of the human brain enhances similarities in the neural dynamics and thus, it could facilitate the creation of a sensorimotor coupling between individuals. These results thus encourage to investigate further inter-brain relationships while drawing a distinction between the “residual synchronizations” due to the sharing of phylogenetic information and common cultural knowledge [51], and the synchronizations related to the brain-to-brain coupling created by the exchange of information through the environment [52]. These two phenomena are not independent and a promising endeavor will be the investigation of their causal relationships.

### 3. Conclusion

To conclude, the nascent social neuroscience could be taken as a new theoretical and experimental workspace in the study of complex systems coupling [69]. Previous studies have already demonstrated the theoretical possibility for dynamical modeling of complex social behavior [70] and sensorimotor coupling in agents [71]. In parallel, neurobiological models have also been proposed to adopt a dynamical and developmental account of socio-cognitive functions at the neural level [72], [73]. The hyperscanning technique starts to provide evidence of the relationships between neural dynamics and social coordination dynamics [74]. Our findings encourage the development of a computational social neuroscience through the extension of existent models at an inter-individual level. It could provide new insights about the neurobiological mechanisms underlying social cognition and related pathologies. Indeed, while individual brain simulations are starting to provide new paths for the understanding of brain lesions [39], growing number of studies describe structural and functional differences in autism [75]–[77] and schizophrenia [78], [79]. Inter-individual neurocomputational models combined with hyperscanning experiments may help in the future to approach these cases of self-other dysfunction.

## Supporting Information

Figure S1 **The connectome.** (A) Rear view. (B) Right view. (C) Top view. (D) Connectivity matrix and legend.
(TIF)

Figure S2 Analysis of the relationship between the low-frequency rhythms observed after the transition produced by increasing the C_intra_ control parameter and the axonal velocity.
(TIF)

Figure S3 Evolution of the inter-brain synchronization over the whole control parameters space in the gamma (A) and alpha (B) frequency bands. The white line delimits the zone where the Mahalanobis distance to real data, computed with PLV and h-PLV matrices in the gamma band, is inferior in the real anatomy than for same shuffled version.
(TIF)

Video S1 Example of simulation where C_intra_ varies continuously across time. Selected signals at both sources and scalp level are represented with their power-spectrum. The bottom part shows the non-linear evolution of the main frequency peak expressed in the signals versus C_intra_.
(MOV)
