# Supplementary figures and images for: Anatomical Connectivity Influences both Intra- and Inter-Brain Synchronizations
Source: PLoS One. 2012 May 10;7(5):e36414. doi: 10.1371/journal.pone.0036414 (PMC3349668; doi:10.1371/journal.pone.0036414)

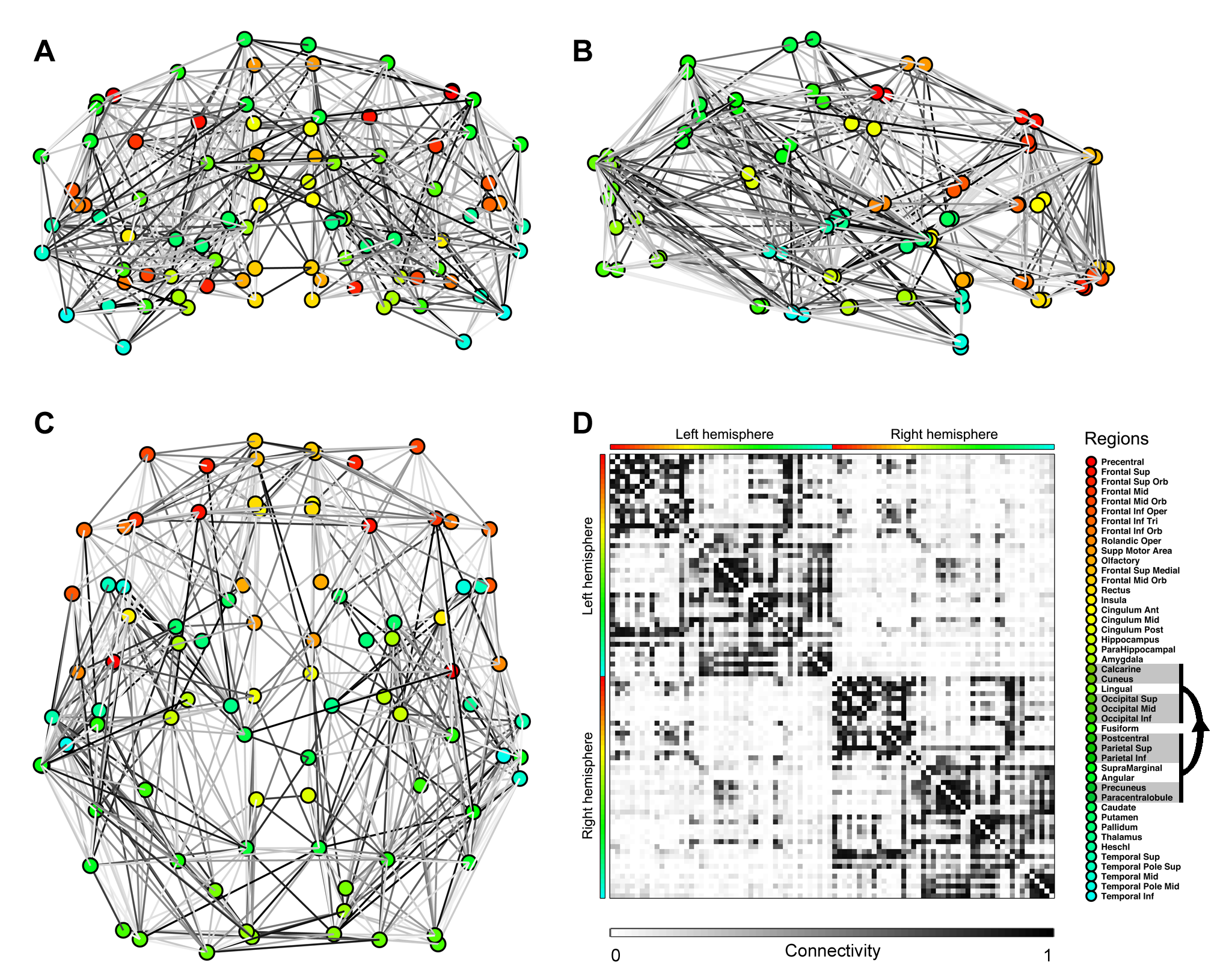

Supplement: Figure S1 — The connectome. (A) Rear view. (B) Right view. (C) Top view. (D) Connectivity matrix and legend. (TIF) [file pone.0036414.s001.tif]

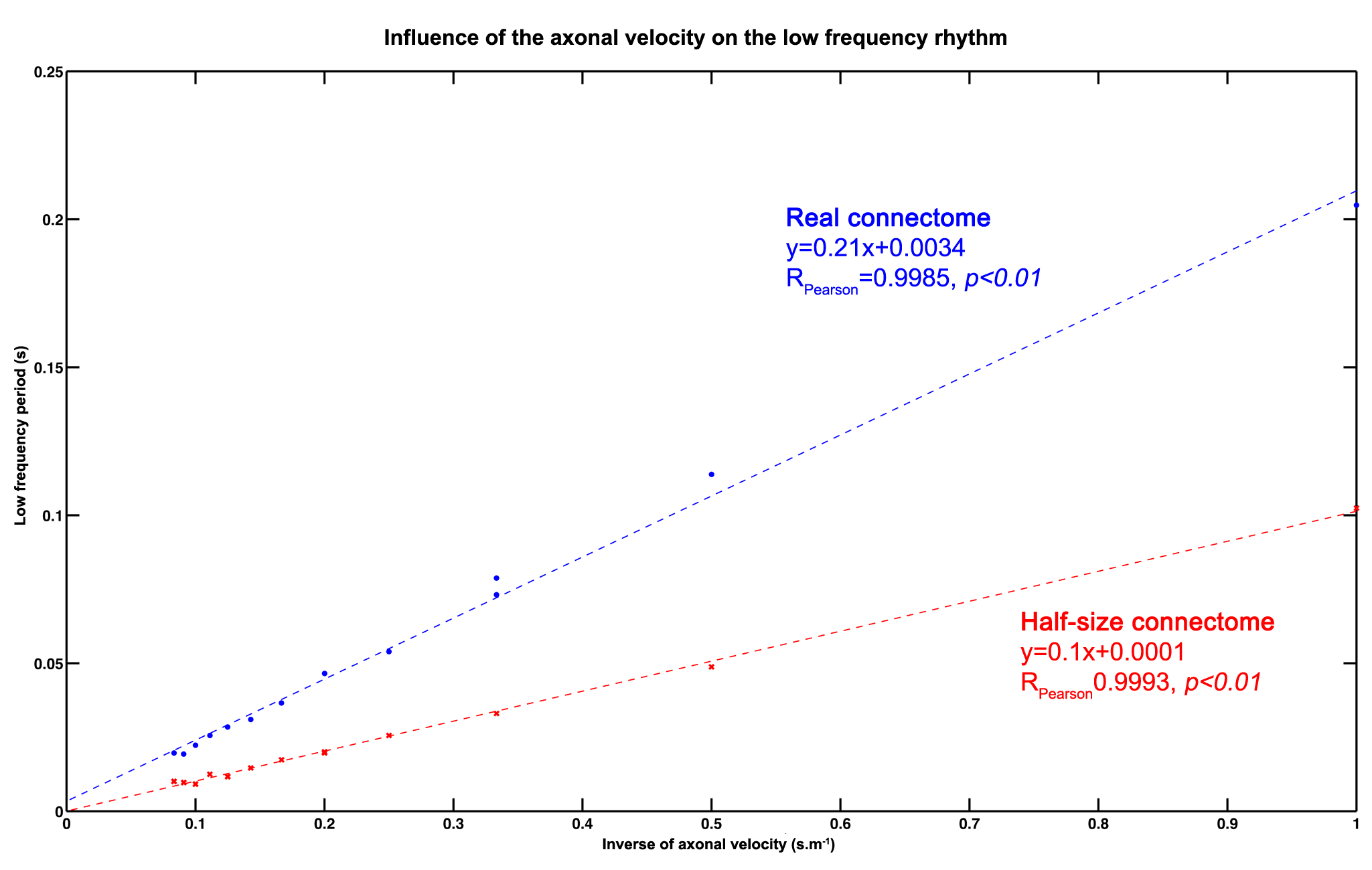

Supplement: Figure S2 — Analysis of the relationship between the low-frequency rhythms observed after the transition produced by increasing the Cintra control parameter and the axonal velocity. (TIF) [file pone.0036414.s002.tif]

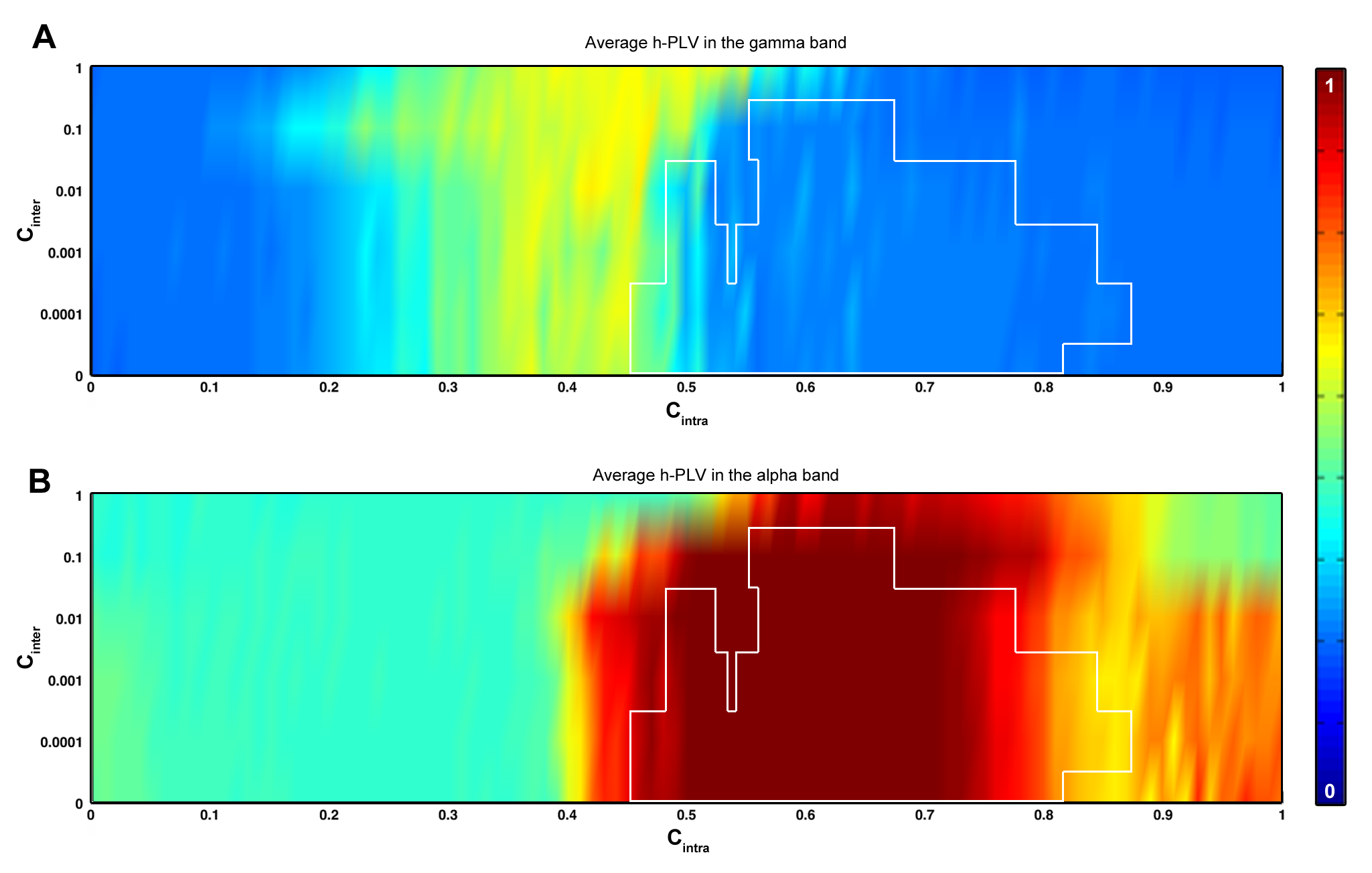

Supplement: Figure S3 — Evolution of the inter-brain synchronization over the whole control parameters space in the gamma (A) and alpha (B) frequency bands. The white line delimits the zone where the Mahalanobis distance to real data, computed with PLV and h-PLV matrices in the gamma band, is inferior in the real anatomy than for same shuffled version. (TIF) [file pone.0036414.s003.tif]
